# circHMGCS1–016 reshapes immune environment by sponging miR-1236-3p to regulate CD73 and GAL-8 expression in intrahepatic cholangiocarcinoma

**DOI:** 10.1186/s13046-021-02095-2

**Published:** 2021-09-15

**Authors:** Ya-Ping Xu, Ze-Ning Dong, Si-Wei Wang, Yi-Min Zheng, Chi Zhang, Ying-Qun Zhou, Yu-Jie Zhao, Yan Zhao, Feng Wang, Rui Peng, Mao-Chun Tang, Dou-sheng Bai, Xiao-Yong Huang, Chuan-Yong Guo

**Affiliations:** 1 Department of Gastroenterology, Shanghai Tenth People’s Hospital, Tongji University School of Medicine, Shanghai, 200072 China; 2 Xiangya Medical College, Central South University, Changsha, Hunan 410008 P. R. China; 3 Ministry of Education, Liver Cancer Institute, Zhongshan Hospital, Fudan University, Key Laboratory of Carcinogenesis and Cancer Invasion (Fudan University), Shanghai, 200032 P. R. China; 4 Department of Hepatobiliary and Pancreatic Surgery, Subei People’s Hospital, Clinical Medical School, Yangzhou University Affiliated Hospital, Yangzhou, China

**Keywords:** Intrahepatic cholangiocarcinoma, circHMGCS1–016, miR-1236-3p, GAL-8, CD73

## Abstract

**Background:**

Accumulating evidence indicates that circRNAs may serve as essential regulators in the progression of several human cancers, but the function and mechanism of circRNAs in intrahepatic cholangiocarcinoma (ICC) are largely unknown.

**Methods:**

RNA-seq was used to assess differentially expressed circRNAs between 4 ICC and peritumor tissues. Quantitative RT-PCR and in situ hybridization were used to determine the circHMGCS1–016 expression in ICC tissues. The function and mechanism of circHMGCS1–016 were further identified via in vivo experiments. The clinical characteristics and prognostic significance of circHMGCS1–016 were analyzed by a retrospective study. The functions of circHMGCS1–016 were assessed via modifying circRNA expression in ICC cells. Moreover, the molecular mechanisms of circHMGCS1–016 in ICC cells were explored by circRNA precipitation, miRNA immunoprecipitation, SILAC and luciferase reporter assays.

**Results:**

We identified that compared with peritumor tissues, ICC tissues expressed hsa_circ_0008621 (circHMGCS1–016) high by RNA-seq, which was further identified by qRT-PCR and in situ hybridization. Moreover, the expression of circHMGCS1–016 was revealed to be associated with survival and recurrence of ICC patients. By regulating circHMGCS1–016 expression, we found that elevated circHMGCS1–016 promoted ICC development both in vitro and in vivo. By SILAC and circRNA-pull down, we demonstrated that circHMGCS1–016 induced ICC cell invasion and reshaped the tumor immune microenvironment via the miR-1236-3p/CD73 and GAL-8 axis. In ICC tissues, we uncovered that a high level of circHMGCS1–016 was positively associated with CD73 and GAL-8 expression and negatively related to the CD8^+^ T cells infiltration, which was further validated by establishing a humanized mouse tumor model. Importantly, we displayed that ICC patients with high levels of circHMGCS1–016 in tumor tissues benefited less from anti-PD1 treatment compared to those with low levels of circHMGCS1–016.

**Conclusions:**

CircHMGCS1–016 is a forceful contributor in ICC development and immune tolerance via miR-1236-3p/CD73 and GAL-8 axis. CircHMGCS1–016 can be explored as a new potential biomarker and therapeutic target for PD1-resistant ICC.

**Supplementary Information:**

The online version contains supplementary material available at 10.1186/s13046-021-02095-2.

## Background

Intrahepatic cholangiocarcinoma (ICC) is the second most common primary liver malignant tumor, with a poor survival prognosis and an increasing incidence in the past three decades [1]. Hepatic resection is currently the mainstay of curative-intent treatment for ICC, but the resectable rate and cure rate are still very low [2]. Moreover, the prognosis for hepatectomy is unsatisfactory due to a high incidence of local recurrence and distant metastases. And the high invasiveness of ICC makes the tumor prone to multifocality, lymph node and vascular invasion, resulting in poor overall survival (OS) after resection [3, 4]. The locoregional and systematic therapies may benefit ICC patients, especially in patients with advanced tumors. Unfortunately, there is little conclusive evidence about efficacy [5]. Based on these results, it is urgent to develop novel, more effective and personalized treatment strategies in ICC.

ICC is characterized by inflammation and tumor immunosuppressive microenvironment, which has a lavish desmoplastic stroma, including a large number of non-immune and immune cells [6]. These cell types can predict OS and recurrence in ICC patients [7]. Indeed, activation of inflammatory pathways and overproduction of cytokines were found in 38% of ICCs by integrative genomic analysis [8]. Moreover, the neutrophil-to-lymphocyte ratio (NLR) was revealed to be a prognostic marker in ICC and suggested to serve as an indicator of therapeutic choices, especially in immunotherapy for ICC patients [9]. Immune checkpoint molecules (ICM), which serve as a host-dependent immune response to fulfil a barrier against the initiation and full-blown state of cancer, was now authenticated to be targets of immunotherapy by the rapid-fire clinical success harnessing the immune checkpoint blockade (ICB) to treat cancers [10, 11]. Intriguingly, limited observations found that several important ICMs, such as PD-L1, B7-H4 and GAL-9, are also highly expressed in a subset of ICC cells, which suggests that although ICB-based immunotherapy may be effective against ICC, it has not been extensively studied so far [12]. Given the remarkable success of ICB therapy in many other solid tumors, there is an urgent need to further understand the immune microenvironment of ICC. Circular RNAs (circRNAs) are a fresh style of endogenous regulatory RNAs with a circular covalently bonded structure [13]. Although circRNAs were first discovered in eukaryotes in 1979, due to advances in genome sequencing, RNA sequencing, and bioinformatics, it was not until recent years that the production and functional mechanisms of circRNAs were initially revealed [14]. So far, thousands of circular RNAs with tissue and cell specificity have been identified from various species. CircRNAs were revealed to achieve their functions mainly through sponging microRNAs (miRNA) to regulate protein production [15]. Remarkably, recent studies uncovered that circRNAs played an outstanding role in the initiation and progression of cancer and could serve as special molecular markers for cancer diagnosis and treatment [16]. For example, circTRIM33-12 impeded hepatocellular carcinoma (HCC) progression via sponging miRNA-191 [17], while circMET promotes the progress of HCC by reshaping the immune microenvironment [18]. In ICC, circRNA SMARCA5 was demonstrated to correlate with better clinical tumor features and prognosis and more sensitivity to chemotherapy [19]. Recently, the roles of circRNAs in tumor immune microenvironment (TME) and immunotherapy have attracted much attention. For instance, cancer cell-derived exosomal circUHRF1 contributed to tumor immunosuppression by inducing NK cell dysfunction and driving anti-PD1 resistance in HCC [20]. Up to now, the study of circRNA in ICC is extremely limited, and the expression and roles of circRNA in ICC need further exploration.

CD73 (ecto-5′-nucleotidase), a cell surface glycosylphosphatidylinositol anchored glycoprotein, is essential for the generation of extracellular adenosine from 5′-adenosine monophosphate (5′-AMP) [21]. CD73 has been identified as a novel immune checkpoint associated with adenosine metabolism that promotes tumor progression by suppressing anti-tumor immune response [22]. GAL-8, a member of -glycan-binding protein family, has also been proposed to play immunosuppressive roles via inducing apoptosis of activated T cells, including Th17 cells, and promoting differentiation of immunosuppressive Tregs [23]. Here we reported that circHMGCS1–016 is frequently upregulated in ICC. Using SILAC and circRNA-pull-down assay, we defined that circHMGCS1–016 promoted ICC cell invasion and reshaped the TME via miR-1236-3p/CD73 and GAL-8 axis. Clinically, we identified that up-regulation of circHMGCS1–016 related to the poor clinical prognosis and high recurrence rate in ICC. By establishing a humanized mouse tumor model and analyzing retrospective data from 12 ICC patients, we demonstrated that ICC with a high level of circHMGCS1–016 showed ineffectively responds to anti-PD1 therapy (Opdivo). Thus, we discovered a powerful immunosuppressive circRNA, which provided a new biomarker for the prediction of prognosis and effective treatment in ICC.

## Methods and materials

### Cell cultures and transfection

ICC cell lines, RBE, HCCC-9810, HUCCT1 and QBC939, were stored in our lab [24]. All cell lines were routinely cultured in RPMI1640 (Gibco, Waltham, MA, USA) containing 10% fetal bovine serum (Gibco, Waltham, MA, USA) and 100 u/ml of penicillin/streptomycin (Yeasen, Shanghai) at 37 °C in a humidified incubator with 5% CO_2_.

The shcircHMGCS1–016, circHMGCS1–016, shmiR-1236-5p, miR-1236-5p, shCD73 and shGAL-8 lentiviral vectors were obtained from Genomeditech company (Shanghai, China). The circHMGCS1–016 shRNA-1 target sequence is 5′-ATAGCAACTGAGGGCTTCGTG-3′ and the shRNA-2 target sequence is 5′-AACTGAGGGCTTCGTGGGACA-3′. The lentiviral plasmid was transfected into ICC cells as our previous study [18]. The down or up efficiency of transfected cells was proven by quantitative real-time polymerase chain reaction (qRT-PCR) or western blot. Other target sequences were listed in Supplementary Table 1.

### Tissue microarray, immunohistochemistry, in situ hybridization, multiplex immunofluorescence, patients and follow-up

The tissue microarray was constructed as described in our previous study [24]. Rabbit polyclonal to human CD73 antibody (1:1000, ab237757, Abcam, USA), Rabbit monoclonal [EPR3610] to human GAL-8 antibody (1:1000, ab92742, Abcam, USA) and CD4 (1:1000, ab203034, Abcam, USA) were used in immunohistochemistry (IHC). All images were captured by the Leica QWin Plus v3 software. The intensity of positive staining was measured as described [25]. The detailed information on antibodies was listed in Supplementary Table 2.

In situ hybridization was performed previously with minor modification and described in supplementary materials and methods [17, 26]. Specimens were collected from 135 patients with ICC who underwent radical resection at the Fudan University Liver Cancer Institute (Shanghai, China) from 2006 to 2008. Ethical approval was confirmed by the Zhongshan Hospital Research Ethics Committee and written informed consent was acquired from each patient (SYXK-2018-034). Following-up data were ended by March 2014, and the following-up median time was 62 months (range 4–121 months).

Multiplex immunofluorescence was performed using a previous protocol following the above steps [27]. Slides were incubated with the primary CD73 (Abcam, Clone EPR23048–139; 1:200), CD4 (Abcam, Clone EPR6855; 1:150), CD8 (Abcam, Clone EP1150Y; 1:450), GAL-8 (Abcam, Clone EPR4857; 1:500) antibodies for 1 h at 37 °C. Then, incubation with BrightVision poly-HRP-anti-Ms/Rb/Rt IgG (DPVO999HRP) at 37 °C for 10 min. The subsequent experiments are conducted according to the instructions.

### The qRT-PCR, western blot, invasion, metastasis, proliferation, and immunofluorescence assay

The qRT-PCR, western blot, invasion, metastasis, proliferation, and immunofluorescence assays were done according to our previous report as described [28].

### circRNA sequencing and analysis

The circRNA sequencing was performed by the Majorbio Biotechnology Co., Ltd. (Shanghai, China). Total RNA was extracted from 4 ICC and paired para-cancerous tissues using Trizol reagent (Invitrogen, USA). The ribo-zero-magnetic-kit (Epicentre, Paris, France) was used to remove ribosomal RNA from the samples. The samples were then incubated for 1 h at 40 °C with RNase R (Illumina, San Diego, CA, USA) to remove linear RNA. Subsequently, the TruSeq RNA LT Sample Prep Kit V2 (Illumina, San Diego, CA, USA) was used to prepare RNA-seq libraries.

### SILAC (stable isotope labeling by amino acids in cell culture)

RBE-control and RBE-circHMGCS1–016 cells were maintained and labeled in SILAC kits (Invitrogen, USA). After cell harvest, equal numbers of cells from two groups were mixed and crude proteins were extracted. The resulting peptides were analyzed by nano-HPLC-MS/MS on Q Exactive mass spectrometer (Thermo Fisher Scientific, USA). The MS/MS data were searched by using MaxQuant with an integrated Andromeda search engine. Tandem mass spectra were searched using the UniProtKB database concatenated with reverse decoy database and protein sequences of common contaminants.

Gene Ontology (GO) annotation proteome was derived from the UniProt-GOA database (http://www.ebi.ac.uk/GOA/). Kyoto Encyclopedia of Genes and Genomes (KEGG) database was used to annotate protein pathways. Domain annotation was performed by using InterProScan on InterPro domain database via Web-based interfaces and services.

### Co-culture assay, chemokine chip and enzyme-linked immunosorbent assay (ELISA)

A co-culture assay with ICC cells and T cells was performed as described [29]. 1 × 10^4^ RBE-control and RBE-circHMGCS1–016 cells were incubated with the conditioned medium in the presence of 50 μM 5′-AMP for 4 h at 37 °C. Human peripheral blood mononuclear cells (PBMCs) labeled with 5 μM carboxyfluorescein diacetate succinimidyl ester (CFSE) were then incubated at 5 × 10^5^ with the above conditioned medium, and 1 μg/ml anti-CD3 mAb for 72 h. The CFSE dilution was measured by flow cytometry by gating on CD8^+^ or CD4^+^ cells.

After 72 h of coculture, the supernatants were collected and analyzed using the Human XL Cytokine Array Kit (R&D Systems, Inc. Minneapolis, USA). The GAL-8, IL-6, IL-2 and IFN-γ concentration in the serum from the peripheral blood of patients with ICC and in the supernatants of different ICC cell lines were measured using an ELISA (RayBiotech, Norcross, GA) according to the manufacturer’s instructions.

### Dual luciferase reporter assay

The mutant luciferase reporter vectors were generated using a mutagenesis kit (Qiagen, CA, USA) according to the manufacturer’s instructions. Plasmids were transiently transfected into 293 T cells, lysed and collected after 48 h via centrifuge at about 15,000 rpm for 3–5 min and supernatants were collected.

Luciferase detection was performed according to the instruction with a measurement time of 10 s and interval of 2 s. For the assay, 20 μl of sample and 20 μl of firefly luciferase assay reagent were gently mixed 2–3 times, and relative light units (RLU) were assessed with cell lysis buffer with a blank control. This was repeated with Renilla luciferase assay reagent, and degree of reporter gene activation was determined by the ratio of both RLU values.

The 3′-UTRs of the circHMGCS1–016 potential target genes were amplified using PCR and cloned into a pGL3 vector. This construct (2 ng) was co-transfected with 2 ng of pRL-TK plasmid. Forty-eight hours after transfection, luciferase activity was measured and normalized. Alternatively, cells were co-transfected with 200 ng of luciferase plasmids, 2 ng of pRL-TK and 20 nM of miRNA/the inhibitor of circHMGCS1–016.

### Human CD34^+^ cell isolation and humanized mice establishment

Cord blood samples were obtained from the pregnant women giving birth to newborns, with written consent obtained from the donors, and following the ethical guidelines of Obstetrics and Gynecology Hospital of Fudan University and Zhongshan Hospital of Fudan University, China (SHDSYY-2014). According to manufacturer’s instructions, human CD34^+^ cells were isolated and purified using CD34 MultiSort Kit (Miltenyibiotec) under sterile conditions. The purity of the CD34^+^ cells was identified by flow cytometry. Humanized NSG mice were established as described in reference [30]. In brief, 3 weeks old NSG mice received sublethal irradiation (360 cGy; X-RAD 320 irradiator), followed by intrahepatic injection of 1 × 10^5^ human Cord blood-derived CD34^+^ cells. 12 weeks later, human immune cells of the humanized NSG mice reconstitution were determined by flow cytometry. Only when human CD45^+^ cells of humanized NSG mice were ≥ 25% in total circulating CD45^+^ cells, the humanized models were considered successfully established and used for subsequent experiments.

### In vivo tumor growth and metastasis assays

The in vivo tumor growth assays were performed using B57/C6 mice. Mice were acquired from the Shanghai Institute of Material Medicine and were fed in a pathogen-free environment. ICC cells were injected subcutaneously into mice with a 27-gauge needle. Tumor sizes were calculated according to the previous report [18]. This ethical approval was obtained from the Research Ethics Committee of Shanghai Tenth People’s Hospital (SHDSYY-2014).

### Statistical analysis

Statistical analysis was performed with SPSS 16.0 software (SPSS). For the immunohistochemical markers, the cut-off for defining the subgroups was the median value. Values are expressed as the mean ± standard deviation. The χ^2^ test and Student’s t-test were used for comparisons between groups. OS and the recurrence were defined as previously described [24]. Kaplan-Meier survival estimates, and Cox’s proportional hazards regression model were performed to assess prognostic significance and factors. All tests were two-tailed, and *p* < 0.05 was considered statistically significant.

## Results

### High endogenous circHMGCS1–016 level in ICC correlates with poor survival and post-resection outcome

**Fig. 1:**
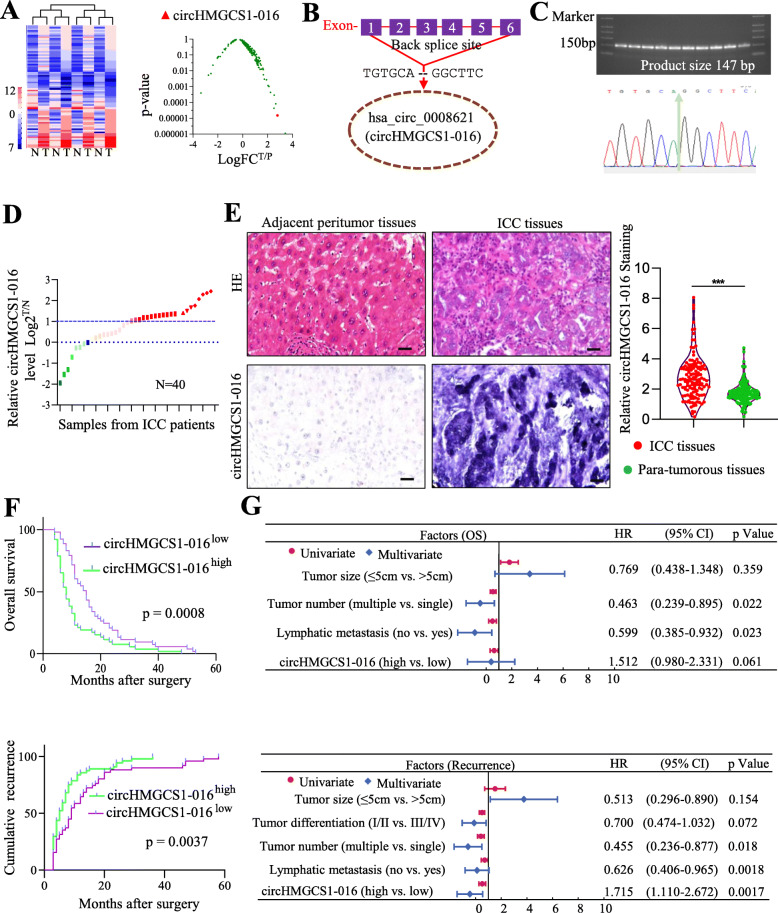
Elevated circHMGCS1–016 in ICC tissues and its clinical implication for ICC patients. **A.** The heatmap of circRNA sequencing showed the top up- and down-regulation of circRNAs between ICC and adjacent nontumor tissues (left). Volcano plot showed the differentially expressed circRNAs and circHMGCS1–016 was among the top 3 up-regulated ones (right);**B.** Schematic illustration of circHMGCS1–016; **C.** RT-PCR amplified the circHMGCS1–016. Electrophoresis showed molecular weight of circHMGCS1–016 and the sequence of back splice site was also presented; **D.** The differential expression of circHMGCS1–016 in ICC tissues and adjacent nontumor tissues of 40 patients as indicated. Data are representative of 3 independent tests; **E.** In situ hybridization analysis showed that ICC samples had higher expression of circHMGCS1–016 than that in matched peri-tumor samples (Bar = 200 μm). The scatter diagram showed the expression of circHMGCS1–016 in ICC and nontumorous tissues (*** *p* < 0.001); **F.** Kaplan-Meier analysis of OS and recurrence in 135 ICC patients according to circHMGCS1–016 expression; **G.** Univariate and multivariate analyses of factors associated with OS and recurrence

Recently, increasing evidence shows the abnormal circRNA expression plays a vital role in several cancers [16]. However, the differential circRNA profiles between dysplastic nodules and adjacent tumor tissues of ICC are still lacking. Using whole-genome circRNA-seq analysis, we identified 76 differentially expressed circRNAs, including 37 up-regulation and 39 down-regulation, between ICC and adjacent tumor tissues by high stringency cut-off (Log_2_^T/*N*^ ≥ 2 and FPKM ≥10 in tumor cells) (Fig. 1A). Among them, five randomly selected circRNAs in the paired tumor and peritumor tissues from the four patients were validated by qRT-PCR. (Fig. S1A and B). Of particular interest is the elevated expression of circHMGCS1–016, derived from the *HMGCS1* gene and locating at chromosome 5p12, a cancer susceptibility region [31](Fig. 1B and C). The expression of circHMGCS1–016 was further verified by qRT-PCR in ICC and paired peritumor tissues from 40 patients (Fig. 1D). Collectively, these results reveal a positive correlation between ICC and the up-regulation of circHMGCS1–016.

Given that circHMGCS1–016 is one of the highly elevated circRNAs in ICC through genome-wide screening, we next investigated the clinical relevance by examining whether the endogenous circHMGCS1–016 level could predict the clinical outcomes of ICC patients. Semi-quantitative in situ hybridization microarray analysis showed that the level of circHMGCS1–016 in ICC tissues is higher than that in adjacent tissues (Fig. 1E, *p* < 0.001). Furthermore, compared with patients with low levels of circHMGCS1–016, patients with higher expression of circHMGCS1–016 in tumor tissues showed significantly shorter survival time and higher cumulative recurrence rate after radical resection. (Fig. 1F, *p <* 0.001). Importantly, univariate and multivariate analyses indicated that the circHMGCS1–016 level was an independent prognostic indicator for ICC patients’ cumulative recurrence (Fig. 1G). Together, these results indicate that elevated expression of circHMGCS1–016 is a driving factor in the progression of ICC.

### circHMGCS1–016 drives ICC development in cell culture and in vivo

**Fig. 2:**
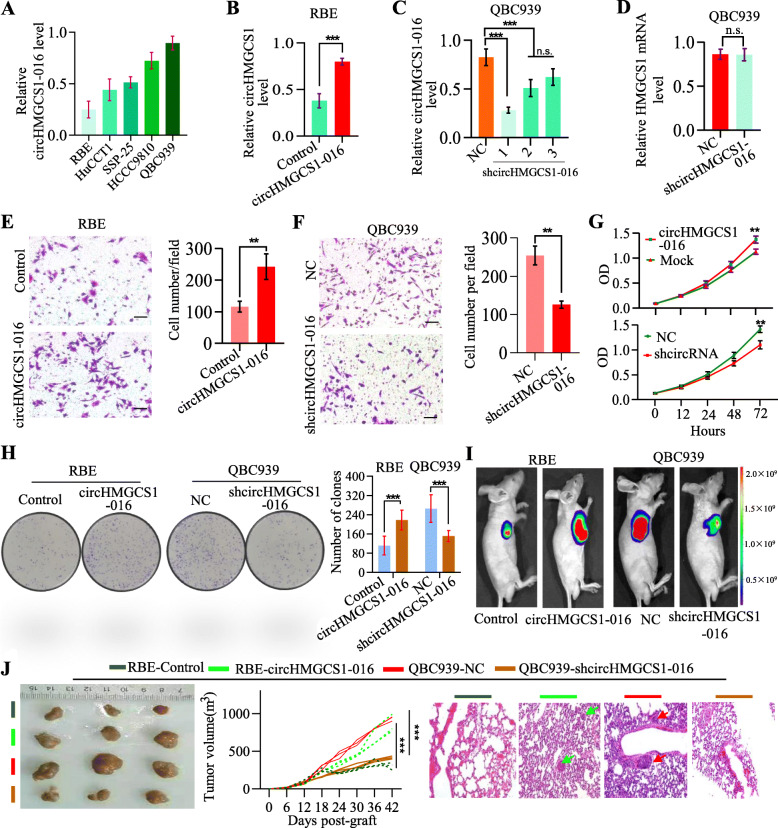
Elevated circHMGCS1–016 promotes ICC progression. **A**. The expression of circHMGCS1–016 in ICC cells was detected by qRT-PCR; Data are representative of 3 independent tests; **B**. The efficacy of circHMGCS1–016 overexpression in RBE cells was analyzed by qRT-PCR; Data are representative of 3 independent tests (***p < 0.001); **C**. The efficacy of circHMGCS1–016 interference in QBC939 cells was analyzed by qRT-PCR; Data are representative of 3 independent tests (* *p* < 0.05, *** *p* < 0.001); **D**. The circHMGCS1–016 interference in QBC939 did not influence the HMGCS1 mRNA expression; Data are representative of 3 independent tests (n.s. *p* > 0.05); **E and F.** Invasion assay was used to detect the invasion ability of ICC cells with different circHMGCS1–016 level (Bar = 200 μm); Data are representative of 3 independent tests (**p < 0.01); **G**. CCK-8 assay showed that the circHMGCS1–016 is positively associated with the proliferation ability of ICC cells; Data are representative of 3 independent tests (** *p* < 0.01); **H.** The ability of colony formation was stronger in ICC cells with higher level of circHMGCS1–016. Data are representative of 3 independent tests (***p < 0.001); **I**. Representative bioluminescence images of subcutaneous xenotransplanted tumors in mice at day 42 after inoculation with ICC cells. The color scale bar depicts the photon flux emitted from the mice (*n* = 6). **J**. Tumorigenesis of RBE-control, RBE-circHMGCS1–016, QBC939-NC and QBC939- circHMGCS1–016 cells in nude mice, and the tumor burden of RBE-circHMGCS1–016 and QBC939-NC cells were larger than those of their control groups; and pulmonary metastasis was found in mice implanted ICC cells expressing high level of circHMGCS1–016 (*** *p* < 0.001)

Based on the clinical evidence that ICC has a potential role in tumor metastasis, we first analyzed the expression of circHMGCS1–016 in 5 ICC cell lines (Fig. 2A). circHMGCS1–016 was then stably knocked down in QBC939 cells with high expression of it, while circHMGCS1–016 was stably elevated in RBE cells with low circHMGCS1–016 expression (Fig. 2B and C). Moreover, we found that the circHMGCS1–016 interference did not change the HMGCS1 mRNA level (Fig. 2D). Invasion and proliferation assays showed that down-regulation of circHMGCS1–016 significantly impaired the invasion and proliferation of QBC939 cells. Conversely, up-regulation of circHMGCS1–016 promoted the invasion and proliferation of RBE cells (Fig. 2E-H). Furthermore, the volumes of tumor in the elevated level of circHMGCS1–016 groups were larger than those of tumors expressing a low level of circHMGCS1–016 (Fig. 2I and J). Notably, pulmonary metastasis was easily found in mice implanted cells expressing a high level of circHMGCS1–016 (Fig. 2J) compared to the mice implanted cells with a low level of circHMGCS1–016. Thus, the up-regulation of circHMGCS1–016 promoted ICC cell proliferation, invasion and metastasis both in vitro and in vivo.

### circHMGCS1–016 sponged the miR-1236-3p in ICC cells

**Fig. 3:**
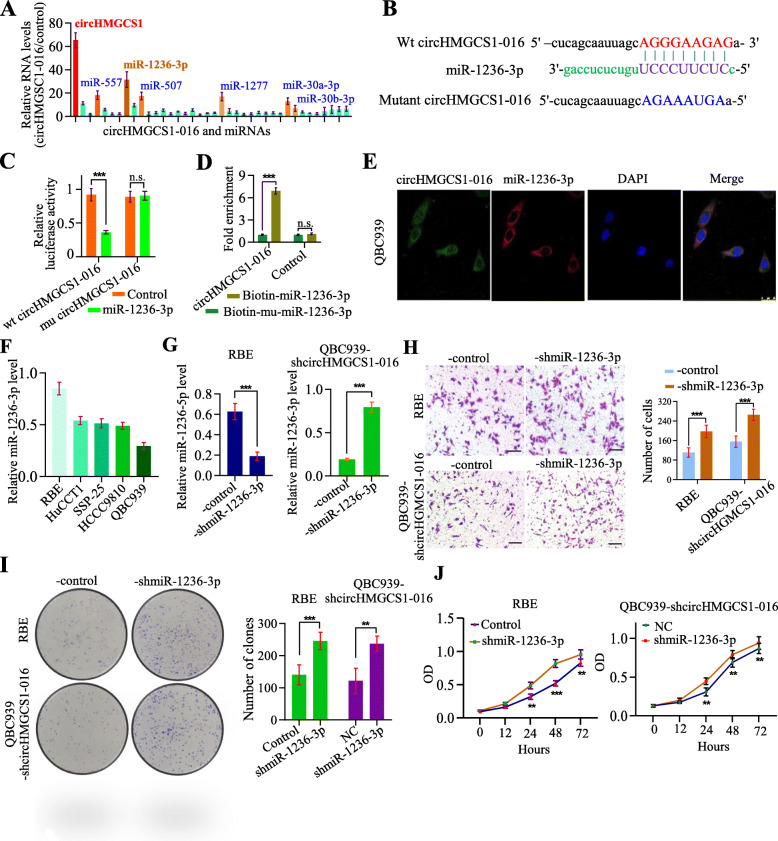
circHMGCS1–016 functions as a sponge for miR-1236-3p. **A**. CircRIP was performed in QBC939 cells using circHMGCS1–016 and negative control probes, and we found a specific enrichment of circHMGCS1–016 and miR-1236-3p compared with the negative control; **B**. Putative binding sites of miR-1236-3p with respect to circHMGCS1–016 were predicated via StarBase v3.0. **C**. The luciferase activity of pLG3-circHMGCS1–016 in the 293 T cells after co-transfection with miR-1236-3p. Data are representative of 3 independent tests (*** *p* < 0.001, n.s. *p* > 0.05); **D**. The level of circHMGCS1–016 in the streptavidin-captured fractions of the QBC939 cell lysates after transfection with biotinylated miR-1236-3p or the negative control (NC). Data are representative of 3 independent tests (*** *p* < 0.001, n.s. *p* > 0.05); **E**. FISH analysis in QBC939 cells showed that circHMGCS1–016 was colocalized with miR-1236-3p in the cytoplasm; **F.** The relative level of miR-1236-3p was measured by qRT-PCR in the ICC cells; Data are representative of 3 independent tests; **G**. The expression of miR-1236-3p was modified in ICC cells; Data are representative of 3 independent tests (*** *p* < 0.001); **H**. The interference of miR-1236-3p in QBC939-shcircHMGCS1–016 and RBE-control cells up-regulated the invasive ability of ICC cells (Bar = 200 μm); Data are representative of 3 independent tests(*** *p* < 0.001); **I**. The interference of miR-1236-3p in QBC939-shcircHMGCS1–016 and RBE-control cells up-regulated the ability of colony formation; Data are representative of 3 independent tests (** *p* < 0.01, *** *p* < 0.001); **J**. CCK-8 assay showed that the interference of miR-1236-3p in QBC939-shcircHMGCS1–016 and RBE-control cells up-regulated the ability of cell proliferation. Data are representative of 3 independent tests (** *p* < 0.01, *** *p* < 0.001)

Considering that circRNAs have been discovered to function as the miRNA sponges [32], we assessed which miRNA would be sponged by circHMGCS1–016 in ICC cells. To this end, we purified circHMGCS1–016-interacting miRNAs by circRIP using circHMGCS1–016-specific probes in QBC939 cells and determined the amount of 36 candidate miRNAs according to the predication by Starbase v3.0 by qRT-PCR. The results showed a high specific enrichment of miR-1236-3p by circHMGCS1–016 compared to the negative control (Fig. 3A).

To further verify that circHMGCS1–016 sponges miR-1236-3p, full-length wild type (WT) circHMGCS1–016 and mutant circHMGCS1–016 without miR-1236-3p binding sites were cloned into the luciferase reporter vector pLG3 (Fig. 3B). A miR-1236-3p mimic significantly reduced luciferase activity of the WT-circHMGCS1–016, but did not significantly reduce the luciferase activity of mutant-circHMGCS1–016(Fig. 3C). Further, pull-down assay with a biotinylated miR-1236-3p mimic showed significant enrichment of circHMGCS1–016 compared to negative controls (Fig. 3D).

In addition, FISH analysis in QBC939 cells showed that circHMGCS1–016 colocalized with miR-1236-3p in the cytoplasm (Fig. 3E). Then, we analyzed the expression of miR-1236-3p in 5 ICC cell lines and modified the expression in RBE cells and QBC939-shcircHMGCS1–016 cells (Fig. 3F-G). In cellular functional assays, decreased expression of miR-1236-3p in ICC cells with a low level of circHMGCS1–016 resulted in the enhanced ability of invasion, proliferation, and clone formation in vitro (Fig. 3H-J). Thus, circHMGCS1–016 acts as a sponge for miR-1236-3p to promote ICC progression.

### circHMGCS1–016 induces immunosuppression in ICC through the miR-1236-3p/CD73 and GAL-8 axis

**Fig. 4:**
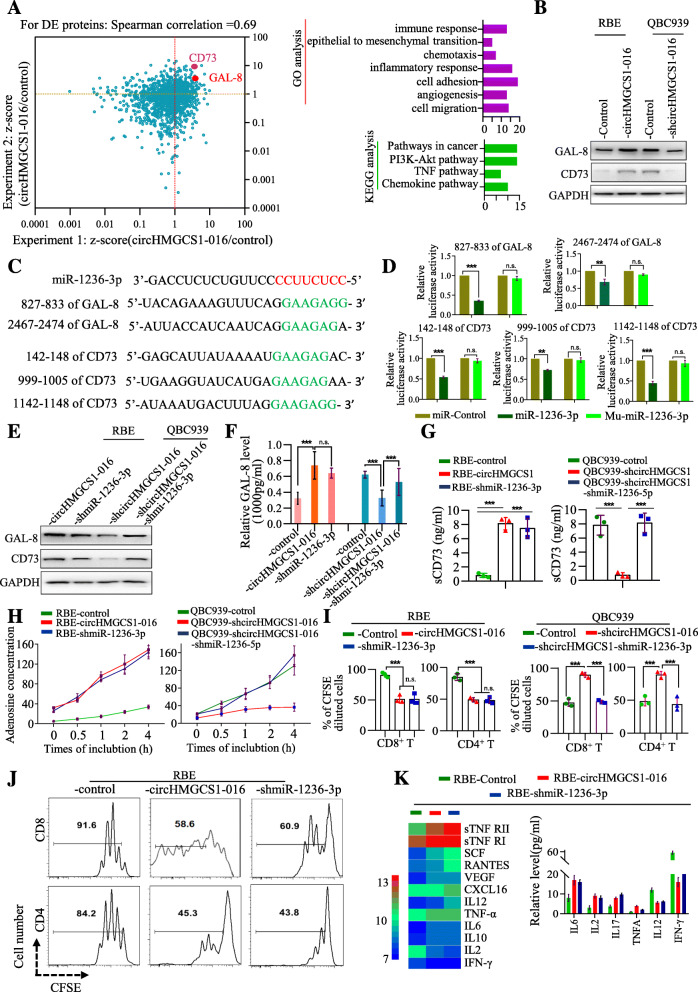
circHMGCS1–016 regulates the miR-1236-3p/GAL-8 and CD73 axis in the ICC cells. **A**. The overlapped differentiated proteins were shown. For differentially expressed proteins (left panel), the GO and KEGG analysis were performed; **B**. Western blot for CD73 and GAL-8 proteins in modified RBE and QBC939 cells; **C**. Putative binding site of miR-1236-3p with respect to GAL-8 and CD73 via StarBase v3.0. **D**. The luciferase activity of pLG3-CD73 or GAL-8 in the 293 T cells co-transfected with miR-1236-3p. Data are representative of 3 independent tests (** *p* < 0.01, *** *p* < 0.001, n.s. *p* > 0.05); **E**. The levels of CD73 or GAL-8 proteins were determined by western blot in the ICC cells with different miR-1236-3p or circHMGCS1–016 expression; **F**. The level of GAL-8 in the supernatant from ICC cells with different miR-1236-3p or circHMGCS1–016 expression was determined; Data are representative of 3 independent tests (*** *p* < 0.001, n.s. *p* > 0.05); **G**. The level of sCD73 in the supernatant from ICC cells with different miR-1236-3p or circHMGCS1–016 expression was determined; Data are representative of 3 independent tests(*** *p* < 0.001); **H**. The level of adenosine concentration in the supernatant from ICC cells with different miR-1236-3p or circHMGCS1–016 expression was determined; Data are representative of 3 independent test; **I and J.** A co-culture showed the supernatant from ICC cells overexpressing circHMGCS1–016 inhibited the CD4^+^ and CD8^+^ T cell proliferation; Data are representative of 3 independent tests (*** *p* < 0.001, n.s. *p* > 0.05); **K.** Chemokine chips and ELISA were employed to determine the different chemokines in the supernatant between RBE-control, RBE-circHMGCS1–016 and RBE-shmiR-1236-3p groups

We further determined the mechanism of miR-1236-3p for favoring ICC development. We performed the SILAC to detect the different proteins between RBE-control and RBE-circHMGCS1–016 cells. Functional analysis revealed that the different proteins were mainly related to those proteins involving in inflammatory response, cell adhesion and migration, angiogenesis, epithelial-mesenchymal transition and immune response. KEGG analysis showed the differentiated proteins mainly correlated with pathways in cancer, chemokine pathway and TNF pathway (Fig. 4A). The proteome analyses supported the notion that circHMGCS1–016 affects multiple cancer and inflammatory response-relating pathways.

Significantly, based on the results of SILAC and the online prediction (targetscan, http://www.targetscan.org/vert_72/), we speculated that CD73 and GAL-8 might be the target of miR-1236-3p in ICC cells (Fig. 4B). We first showed putative binding sites of CD73 and GAL-8 with miR-1236-3p (Fig. 4C). Dual-luciferase reporter assay further showed that luciferase activity was reduced in cells transfected with wt-miR-1236-3p compared to cells transfected with mutant-miR-1236-3p (Fig. 4D).

To better understand the roles of miR-1236-3p in circHMGCS1–016-induced function, we constructed lentiviral vectors expressing miR-1236-3p or shmiR-1236-3p and established stable cell lines. CD73 and GAL-8 expression was upregulated in RBE-circHMGCS1–016 than RBE-shmiR-1236-3p (Fig. 4E) but was downregulated in QBC939-shcircHMGCS1–016 compared to QBC939-shcircHMGCS1–016-shmiR-1236-3p (Fig. 4E). This indicates that CD73 and GAL-8 is a target of miR-1236-3p and circHMGCS1–016 promote ICC progression via miR-1236-3p/CD73 and GAL-8.

Here, we further determined the GAL-8 in the supernatant of ICC cells with different circHMGCS1–016 or miR-1236-3p expression by ELISA and high concentration of GAL-8 was found in cells overexpressing circHMGCS1–016, while low in cells expressing low level of miR-1236-3p level (Fig. 4F), which was consistent with the supernatant sCD73 level in ICC cells (Fig. 4G). Importantly, we established a co-culture system of peripheral blood mononuclear cells (PBMCs) and tumor cells with different circHMGCS1–016 and miR-1236-3p expression levels. As shown in (Fig. 4H-J), RBE cells overexpressing circHMGCS1–016 or low miR-1236-3p levels are positively correlated with high levels of adenosine concentration, while negatively correlated with the proliferation of CD8^+^ T cells and CD4^+^ T cells at different time points. Furthermore, human cytokine arrays were used to detect the cytokine profile in co-culture supernatants. Elevated circHMGCS1–016 expression was associated with low levels of the effector cytokines such as IFN-γ, IL-2, and high levels of immunosuppressive cytokines such as IL-10, IL-6, TNF-α, VEGF, SCF1, sTNFRI and sTNFRII in the supernatants (Fig. 4K). Thus, we conclude that circHMGCS1–016 overexpression induces a compromised immunosuppressive microenvironment in ICC.

### circHMGCS1–016/miR-1236-3p/CD73 and GAL-8 axis induces immunosuppression in ICC

**Fig. 5:**
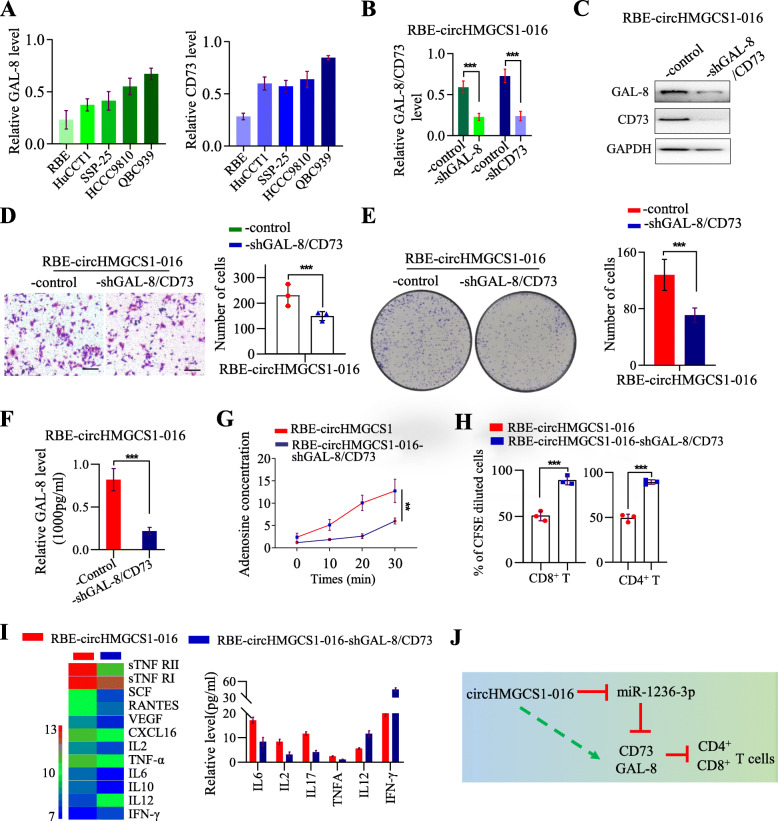
GAL-8 and CD73 interference restraints the function of circHMGCS1–016 in ICC cells. **A**. The relative level of CD73 and GAL-8 was measured by qRT-PCR in the ICC cells; Data are representative of 3 independent tests; **B**. The expression of CD73 and GAL-8 were modified by lentivirus-mediated knockdown in RBE-circHMGCS1–016 cells. Data are representative of 3 independent tests(*** *p* < 0.001); **C**. The efficacy of GAL-8/CD73 interference was analyzed by western blot; **D**. Invasion assay was performed to detect the invasion ability of RBE-circHMGCS1–016 cells and RBE-circHMGCS1–016 cells with CD73 and GAL-8 interference (Bar = 200 μm). Data are representative of 3 independent tests(*** *p* < 0.001); **E**. Colony formation assay was performed to detect the ability of colony formation in RBE-circHMGCS1–016 cells and RBE-circHMGCS1–016 cells with CD73 and GAL-8 interference. Data are representative of 3 independent tests(*** *p* < 0.001); **F**. The level of GAL-8 in the supernatant of RBE-circHMGCS1–016 cells and RBE-circHMGCS1–016 cells with CD73 and GAL-8 interference; Data are representative of 3 independent tests(*** *p* < 0.001); **G**. The level of adenosine concentration in the supernatant of RBE-circHMGCS1_016 cells and RBE-circHMGCS1–016 cells with CD73 and GAL-8 interference; Data are representative of 3 independent tests(** *p* < 0.01); **H**. A co-culture showed the supernatant from RBE-circHMGCS1–016 cells inhibited the CD4^+^ and CD8^+^ T cell proliferation compared to RBE-circHMGCS1–016 cells with CD73 and GAL-8 interference (*** *p* < 0.001); **I**. Chemokine chips and ELISA were employed to determine the different chemokines in the supernatant between RBE-circHMGCS1–016 cells and RBE-circHMGCS1–016 cells with CD73 and GAL-8 interference; **J**. A model for circHMGCS1–016 driven ICC development and established the immune privilege microenvironment

We further identified that circHMGCS1–016 induces immunosuppression via miR-1236-3p/CD73 and GAL-8 axis by interfering with CD73 and GAL-8 expression. Firstly, we determined the CD73 and GAL-8 expression in ICC cell lines and found that both CD73 and GAL-8 are highly expressed in QBC939 cells, while lowly expressed in RBE cells (Fig. 5A), which is positively correlated with circHMGCS1–016 expression. Thus, we interfered with the expression of CD73 and GAL-8 in RBE-circHMGCS1–016 cells (Fig. 5B and C). The invasion assay showed that CD73 and GAL-8 knockdown significantly decreased the invasion of RBE-circHMGCS1–016 cells (Fig. 5D). In colony formation assay, knockdown of CD73 and GAL-8 inhibited colony formation (Fig. 5E). Together, these observations indicate that CD73 and GAL-8 are the downstream molecules of circHMGCS1–016 in ICC.

To better understand the role of CD73 and GAL-8 in circHMGCS1–016-induced immunosuppression, we co-cultured peripheral blood mononuclear cells (PBMC) and circHMGCS1–016 overexpression cells with different expression levels of CD73 and GAL-8. As expected, the GAL-8 and adenosine concentration were down-regulated in the RBE-circHMGCS1–016-shGAL-8 and CD73 group (Fig. 5F and G), and the supernatants of circHMGCS1–016-overexpressing RBE cells restrained seriously the proliferation of CD8^+^ T cells and CD4^+^ T cells compared to the supernatants from the RBE-circHMGCS1–016-shGAL-8/CD73 cells at indicated time points (Fig. 5H). Furthermore, human cytokine arrays showed co-culture supernatants from RBE-circHMGCS1–016 group was associated with lower levels of the effector cytokines such as IL-2, IFN-γ and higher levels of immunosuppressive cytokines such as sTNF RII, sTNF RI, SCF, RANTES, VEGF, CXCL16, IL2, TNF-α, IL-6 and IL-10 in the supernatants compared to RBE-circHMGCS1–016-shGAL-8 /CD73 group (Fig. 5I). Thus, we concluded that circHMGCS1–016 overexpression induced a compromised immunosuppressive microenvironment via miR-1236-3p/GAL-8 and CD73 axis in ICC (Fig. 5J).

### The level of circHMGCS1–016, CD73 and GAL-8 negatively associated with tumor-infiltrating CD8^+^ lymphocytes in ICC

**Fig. 6:**
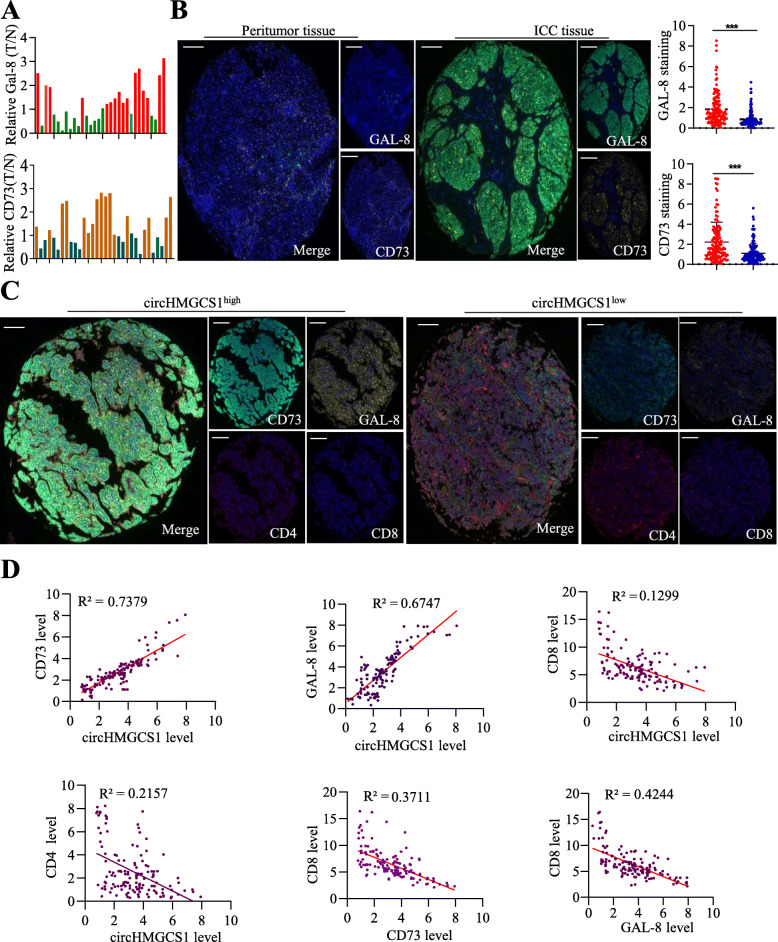
The relationship among circHMGCS1–016, CD73 and GAL-8 in ICC tissues. **A**. CD73 and GAL-8 levels in 40 pairs of ICC and matched adjacent non-tumor tissues. Data was shown as log_2_^(T/N)^; **B**. Representative images for CD73 and GAL-8 staining of ICC and matched adjacent nontumor tissues (Bar = 100 μm); **C**. Representative images for circHMGCS1–016, CD73, GAL-8, CD4 and CD8 staining of ICC tissues (Bar = 200 μm); **D**. Correlation analysis showed the positive relationship between circHMGCS1–016 and CD73(*R*^2^ = 0.7379, *p* = 3.45E-18), circHMGCS1–016 and GAL-8 (*R*^2^ = 0.6747, *p* = 5.87E-16). But circHMGCS1_016 expression was adversely related to the level of CD8 (*R*^2^ = 0.1299, *p* = 6.45E-17) and CD4 (*R*^2^ = 0.2157, *p* = 1.17E-14). The relationship between CD8 and CD73(*R*^2^ = 0.3711, *p* = 1.81E-12), CD8 and GAL-8 (*R*^2^ = 0. 4244, *p* = 2.12E-23) was also analyzed (*** *p* < 0.001)

Here, we further explored CD73 and GAL-8 in ICC samples and found that the expression of CD73 and GAL-8 mRNA in ICC tissue was up-regulated compared with adjacent tissues (Fig. 6A). IHC staining of CD73 and GAL-8 showed that both proteins in ICC tissues are significantly higher than those in the peritumor tissues (Fig. 6B). To validate the function of the miR-1236-3p/CD73 and GAL-8 axis in ICC, we further determined the expression of circHMGCS1–016, CD73, GAL-8, and immune cells in ICC tissues in series TMA (Fig. 6C). Consistent with the results of our above functional studies, we found that the endogenous circHMGCS1–016 level in ICC patients’ tumor tissues was positively correlated with the level of CD73 (*R*^2^ = 0.7379, *p* < 0.0001) and GAL-8 (*R*^2^ = 0.6747, *p <* 0.0001). It was also inversely associated with the level of CD4^+^ T cells (*R*^2^ = 0.2157, *p <* 0.0001) and CD8^+^ T cells (*R*^2^ = 0.1299, *p <* 0.0001) in ICC tissues (Fig. 6D). Additionally, we further analyzed the relationship between CD73, GAL-8 and CD8^+^ T cells and found that CD73 is negatively correlated with CD8^+^ T cells (*R*^2^ = 0.3711, *p <* 0.0001), and so is GAL-8 (*R*^2^ = 0.4244, *p <* 0.0001). Based on the relationship between circHMGCS1–016 and CD8^+^ T cells, we suspect that the circHMGCS1–016 reprogram tumor immune microenvironment through synchronously regulating CD73 and GAL-8 expression.

### circHMGCS1–016 enhances ICC resistance to anti-PD1 therapy

**Fig. 7:**
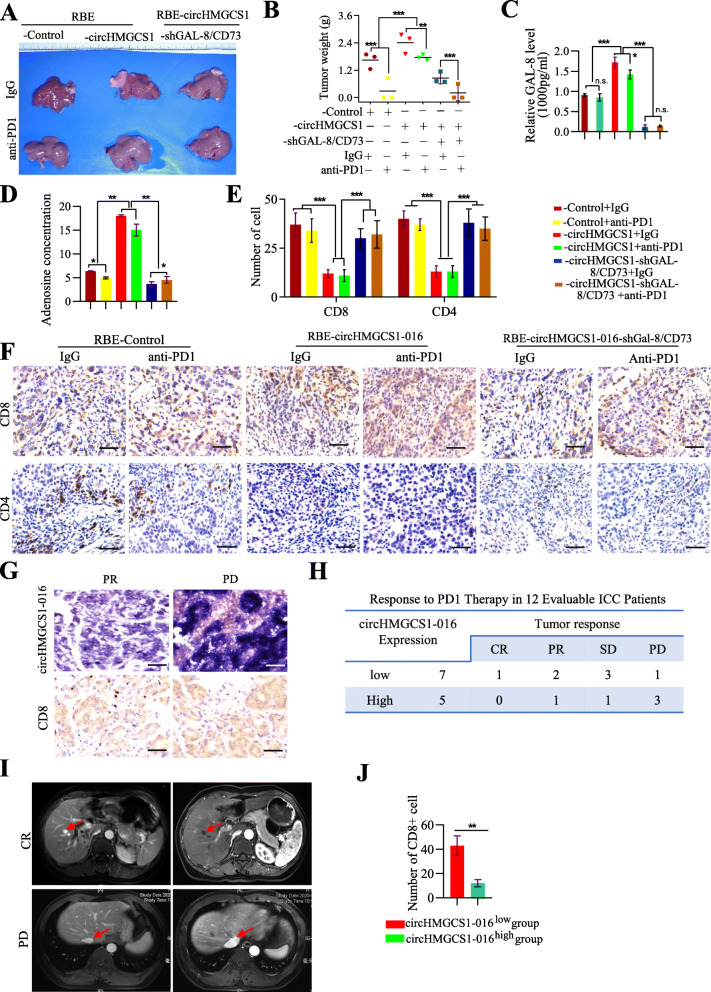
Higher levels of circHMGCS1–016 correlated with resistance to anti-PD1 therapy in mice and ICC patients. **A**. Representative images of the RBE orthotopic ICC tumors from humanized NSG mice (*n* = 3 /group). **B**. Tumor growth volume of the RBE orthotopic planted humanized mice from each group (*** *p* < 0.001, ** *p* < 0.01); **C**. The level of GAL-8 in the serum of the RBE orthotopic ICC tumors from each group; Data are representative of 3 independent tests (*** *p* < 0.001, * *p* < 0.05, n.s. *p* > 0.05); **D**. The level of adenosine concentration in the serum of mice planted with the RBE orthotopic ICC tumors; Data are representative of 3 independent tests(** *p* < 0.01, * *p* < 0.05); **E**. The CD8^+^ and CD4^+^ T cells in the blood of mice planted with the RBE orthotopic ICC tumors (*** *p* < 0.001); **F.** At study endpoint, the CD8^+^ and CD4^+^ T cells in IgG and PD-1 treatment group were analyzed by immunohistochemistry (Bar = 200 μm); **G**. Representative ICC cases from 12 patients who received PD-1 antibody treatment were analyzed by IHC staining for CD8 and circHMGCS1–016 (Bar = 200 μm); **H**. Twelve patients were divided into two groups according to circHMGCS1–016 expression, and patients in CR, PR, SD, and PD were shown in each group. **I**. The efficacy of PD1 antibody immunotherapy was assessed by MRI based on RECIST1.1. **J**. The number of CD8^+^ cells was significantly different between two groups(** *p* < 0.01)

The above results indicate that high levels of circHMGCS1–016 lead to a suppressed immune environment by up-regulating the levels of CD73 and GAL-8, which in turn induces the immune escape of ICC cells. Then, we further studied the anti-tumor efficacy of PD1 antibodies on RBE-control, RBE-circHMGCS1–016 and RBE-circHMGCS1–016-shGal-8/CD73 tumor in humanized mice. We found that PD1 antibodies inhibited the RBE-control and RBE-circHMGCS1–016-shGal-8/CD73 tumors, but not the RBE-circHMGCS1–016 tumors (Fig. 7A and B). Moreover, the serum from mice injected with RBE-circHMGCS1–016 showed a higher level of GAL-8 and adenosine than those in other groups (Fig. 7C and D). Significantly, the PD1 antibodies markedly increased the blood CD8^+^ T cells and CD4^+^ T cells in RBE-control and RBE-circHMGCS1–016-shGal-8/CD73 groups compared to RBE- circHMGCS1–016 and RBE- circHMGCS1–016-shcontrol groups, respectively, while PD1 antibodies treatment could not increase the number of CD8^+^ T cells and CD4^+^ T cells in the RBE-circHMGCS1–016 group compared to IgG antibodies treatment (Fig. 7E). Tissue sections from the RBE-circHMGCS1–016 humanized mice treated by IgG and PD1 antibodies contained fewer tumor-infiltrating CD8^+^ and CD4^+^ lymphocytes than other groups (Fig. 7F).

Furthermore, we analyzed retrospective data from 12 advanced ICC patients receiving PD1 antibody therapy. Representative ICC cases were analyzed by IHC staining for CD8 and circHMGCS1–016 (Fig. 7G). After 6 treatment cycles, MRI was used to evaluate the efficacy. Based on RECIST1.1, one patient achieved a complete response (CR), three patients had partial responses (PR), and four patients remained stable disease (SD) and four patients showed continued progressive disease (PD) at the end of follow-up. Then, circHMGCS1–016 level was measured by in situ hybridization. Among these five patients with tumors expressing a high level of circHMGCS1–016, only one patient had PR, one patient in SD, and three patients in PD. However, among the 7 patients whose tumors expressed low levels of circHMGCS1–016, one patient received CR, two patients were in PR, three patients were in SD and one patient was in PD. (Fig. 7H-I). To further explore the relationship between circHMGCS1–016 and immune escape, we examined the expression of CD8 in 12 cases of ICC tissues. Compared with the tissues of ICC patients sensitive to anti-PD1 therapy, the number of CD8^+^ cells in the tissues of ICC patients resistant to anti-PD1 therapy was significantly reduced (Fig. 7J). In summary, these findings indicate that elevated circHMGCS1–016 expression impedes the response of ICC to anti-PD1 treatment and targeting circHMGCS1–016 might be a promising and effective method to recover the sensitivity of ICC to anti-PD1 therapy.

## Discussion

Increasing studies have reported that aberrant circRNA expression played essential roles in cancer pathophysiologies, such as cell apoptosis, invasion and migration. Recently, emerging evidence has revealed that the dysregulation of circRNAs could promote the tumor progression via reshaping the tumor immune microenvironment. Here, we first identified differentially expressed circRNA profiles between ICC and peritumor tissues from ICC patients and revealed that the circHMGCS1–016 expression in ICC tissues related to the prognosis and recurrence of ICC patients. Importantly, we uncovered that circHMGCS1–016 overexpression negatively regulates anti-tumor immunity through elevating the expression of CD73 and GAL-8 protein simultaneously, which further depressed CD8^+^ T cells and CD4^+^ T cells and reduced the anti-tumor activity of immune cells. By establishing humanized mice, we showed that high level of circHMGCS1–016 could promote anti-PD1 resistance. Thus, we demonstrated that circHMGCS1–016 serves as a promoter in ICC progression and can be a potential biomarker of predicting OS and relapse in ICC– as well as a promising therapeutic target.

Accumulating evidence indicate that dysregulation of circRNAs plays a crucial role in the progression of many cancers. For example, upregulation of circMET has been reported to drive HCC progression by shielding cancer from anti-tumor immunity and fostering tumor resistance to anti-PD1 treatment [18]. A high level of exosomal circUHRF1 induces NK cell exhaustion and causes HCC resistance to anti-PD1 therapy [19], while low level of circTRIM33–12 promoted HCC development by acting as the sponge of miR-191 [17]. In ICC, circ-SMARCA5 was found to be reduced in tumor compared to adjacent tissues, and the level of circ-SMARCA5 was found to be related to the chemotherapy sensitivity in ICC cells [19]. Although new evidence has identified the vital roles of circRNAs in various cancers, there are few reports on circRNAs in ICC. Here, we identified the expression profiles of dysregulated circRNAs in ICC and authenticated elevated circHMGCS1–016 expression as an essential factor in driving ICC progression by multiple lines of clear evidence. An interesting finding in the present study is that we uncovered that circHMGCS1–016 served as a regulator of TME by its impact on the expression of CD73 and GAL-8 proteins. We also verified that a high level of circHMGCS1–016 induced tumors to resist anti-PD1 therapy in NSG mice and ICC patients. These findings collectively elucidate new mechanisms underlying ICC progression induced by circHMGCS1–016 and establish circHMGCS1–016 as a potential and novel target for ICC therapy.

CD73 is a novel immunoinhibitory protein that plays a crucial role in tumor growth and metastasis. Its primary function is to convert extracellular ATP to immunosuppressive adenosine in concert with CD39 in normal tissues to limit excessive immune response [29]. In recent years, CD73 has been defined to be another crucial immune checkpoint molecule. For example, high levels of CD73 have been observed in cancer patients who resist anti-PD1 immunotherapy. Moreover, elevated CD73 has been a vital factor for anti-PD1 therapy resistance in several types of cancer [33, 34]. GAL-8 has also been found to be a potential promoter of immune-regulatory networks that induce chemoattraction and cancer progression at the metastatic niche [23, 35]. Additionally, another study demonstrated that GAL-8 could promote the oncogenic-like transformation of epithelial cells through partial and reversible EMT, accompanied by higher proliferation, migration/invasion, and tumorigenic properties, which indicated that GAL-8 not only impact TME but also indirectly affect the tumor cells [36]. Here, we found that CD73 and GAL-8 proteins are overexpressed in ICC tissues, which are negatively correlated with CD8^+^ T cell infiltration. This conclusion has been further confirmed by in vitro and in vivo studies. This study found that elevated circHMGCS1–016 expression enhanced invasion and colony formation of ICC cells, which might be due to the upregulation of GAL-8 and CD73. Allow for the vital roles of CD73 and GAL-8 in TME, we conclude that the circHMGCS1–016 is a powerful molecule in ICC progression.

## Conclusions

In summary, our findings provide in vitro and in vivo evidence to support the notion that circHMGCS1–016 overexpression serves as a “driver” in ICC development by enhancing the invasiveness of tumor cells and reshaping the TME of ICC via miR-1236-3p/CD73 and GAL-8 axis. These findings reveal the mechanism of circHMGCS1–016 in ICC progression and suggest that circHMGCS1–016 is a potential biomarker that can be used to predict the sensitivity of anti-PD1 therapy. Targeting circHMGCS1–016 may be a promising and effective method to restore the sensitivity of ICC to anti-PD1 therapy.

## Supplementary Information

**Additional file 1: Supplementary Fig. 1.** The Expression of circHMGCS1–016 in ICC Tissues. **A**. The expression of 5 circRNAs randomly selected from the circRNA-seq was investigated in the 4 ICC samples used for small circRNA-Seq by qRT-PCR analysis; **B**. Significant consistency (*R*^2^ = 0.9013, *p* < 0.0001) was found in the results from qRT-PCR and circRNA-Seq (Log_2_^T/N^).

**Additional file 2: Supplementary Table 1.** The primers used in this article.

**Additional file 3: Supplementary Table 2.** The antibodies used in the article.

## Data Availability

All data and materials supporting the findings of this work are available from its supplementary information files and from the corresponding author upon reasonable request.

## Abbreviations

ICC: Intrahepatic cholangiocarcinoma
SILAC: Stable isotope labeling with amino acids in cell culture
OS: Overall survival
TME: Tumor immune microenvironment
NLR: Neutrophil-to-lymphocyte ratio
qRT-PCR: Quantitative reverse transcriptase polymerase chain reaction
GFP: Green fluorescent protein
ICM: Immune checkpoint molecules
ICB: Immune checkpoint blockade
miRNA: microRNA
circRNA: Circular RNA
GO: Gene Ontology
KEGG: Kyoto Encyclopedia of Genes and Genomes
ELISA: Enzyme-linked immunosorbent assay
TMA: Tissue microarray
TNM: Tumor node metastasis
IHC: Immunohistochemistry
RLU: Relative light units
