# Supplementary material for: circHMGCS1–016 reshapes immune environment by sponging miR-1236-3p to regulate CD73 and GAL-8 expression in intrahepatic cholangiocarcinoma
Source: J Exp Clin Cancer Res. 2021 Sep 15;40:290. doi: 10.1186/s13046-021-02095-2 (PMC8442376; doi:10.1186/s13046-021-02095-2)
Supplement: Supplementary file 2 — Additional file 2: Supplementary Table 1. The primers used in this article. [file 13046_2021_2095_MOESM2_ESM.docx]

**Supplementary Table 1. The primers used in this article**

| circRNAs or Genes | Forward (5'--3') | Reverse (5'--3') |
| --- | --- | --- |
| hsa_circ_0000918 | GCTCCAGGTCTTGGCATATT | ACACCCTGTTTCTGGAGCAC |
| hsa_circ_0006137 | TGTTGCACAGAATGGTTCGT | CGCGCTTACATGAAATTTGT |
| hsa_circ_0001258 | TTCAGGCAAAACAGAAAAACA | GTGAGCCTCATCACACAGGA |
| hsa_circ_0119824 | TGCTCCATTTTCTTTGTTTCC | TGCATCTTTCTTCAACATGTCC |
| hsa_circ_0037353 | AGGCAGCACCTCTACCTTCA | GGTGAAACTGACCACAGTGC |
| hsa_circ_0008621 | GAAAAGCACAGAAGAACTTACGC | GGCATGTTGCATATGTGTCC |
| CD73 | GCCTGGGAGCTTACGATTTTG | TAGTGCCCTGGTACTGGTCG |
| GAL-8 | ATCTATAACCCGGTAATCCCGTT | CATGCCCACGTATCACAATCAA |
| GAPDH | CTGGGCTACACTGAGCACC | AAGTGGTCGTTGAGGGCAATG |
