# Supplementary material for: circHMGCS1–016 reshapes immune environment by sponging miR-1236-3p to regulate CD73 and GAL-8 expression in intrahepatic cholangiocarcinoma
Source: J Exp Clin Cancer Res. 2021 Sep 15;40:290. doi: 10.1186/s13046-021-02095-2 (PMC8442376; doi:10.1186/s13046-021-02095-2)
Supplement: Supplementary file 3 — Additional file 3: Supplementary Table 2. The antibodies used in the article. [file 13046_2021_2095_MOESM3_ESM.docx]

**Supplementary table 2. The antibodies used in the article**

| **Antibodies** | **Source** | **Catalog #** |
| --- | --- | --- |
| Anti-CD8 alpha antibody [EPR21769] (mouse) | Abcam | Ab217344 |
| Rabbit polyclonal to Argonaute-2 | Abcam | ab32381 |
| Rabbit polyclonal to CD73 | Abcam | ab168633 |
| Rabbit monoclonal to CD4 | Abcam | ab215206 |
| Rabbit polyclonal to GAPDH | Abcam | ab9485 |
| [Galectin-8 Antibody](https://www.novusbio.com/products/galectin-9-antibody-oti1d12_nbp2-45619) (human) | Novus | [NBP2-45619](https://www.novusbio.com/products/galectin-9-antibody-oti1d12_nbp2-45619) |
| [CD8 alpha Antibody](https://www.novusbio.com/products/cd8-alpha-antibody-yts10518_nb200-578)(human) |  | [NB200-578](https://www.novusbio.com/products/cd8-alpha-antibody-yts10518_nb200-578) |
| [IL6 Antibody (OTI2F4)](https://www.novusbio.com/products/il6r-antibody-oti2f4_nbp2-45414) | Novus | [NBP2-45414](https://www.novusbio.com/products/il6r-antibody-oti2f4_nbp2-45414) |
| Galectin-8 (LGALS8) ELISA Kit | Abebio, Wuhan, China | AE35662MO |
