# Supplementary material for: circHMGCS1–016 reshapes immune environment by sponging miR-1236-3p to regulate CD73 and GAL-8 expression in intrahepatic cholangiocarcinoma
Source: J Exp Clin Cancer Res. 2021 Sep 15;40:290. doi: 10.1186/s13046-021-02095-2 (PMC8442376; doi:10.1186/s13046-021-02095-2)
Supplement: Supplementary file 3 [file 13046_2021_2095_MOESM1_ESM.docx]

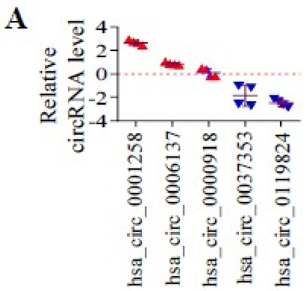

A
Relative circRNA level
4
2
0
-2
-4
hsa_circ_0001258
hsa_circ_0006137
hsa_circ_0000918
hsa_circ_0037353
hsa_circ_0119824

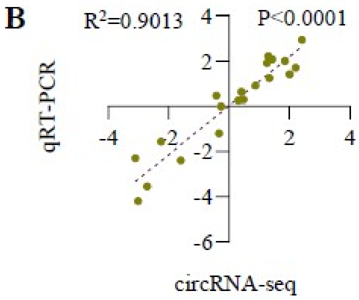

B
R2=0.9013
P<0.0001
qRT-PCR
circRNA-seq
